# Transcriptional regulation of the anti-inflammatory cytokine IL-10 in acquired immune cells

**DOI:** 10.3389/fimmu.2012.00275

**Published:** 2012-08-30

**Authors:** Masato Kubo, Yasutaka Motomura

**Affiliations:** ^1^Division of Molecular Pathology, Research Institute for Biological Sciences, Tokyo University of ScienceNoda, Japan; ^2^Laboratory for Signal Network, Research Center for Allergy and Immunology, RIKEN Yokohama InstituteYokohama, Japan

**Keywords:** epigenetics, interleukin-10, plasticity, regulatory B cells, transcriptional regulation

## Abstract

Although the major role of the immune response is host defense from a wide range of potentially pathogenic microorganisms, excess immune responses can result in severe host damage. The host thus requires anti-inflammatory mechanisms to prevent reactivity to self. Interleukin-10 (IL-10) is a cytokine with broad anti-inflammatory properties involved in the pathogenesis of various diseases. IL-10 was originally described as a T helper (T_H_2) derived cytokine, but further studies indicated that IL-10 is expressed not only by many cells of the adaptive immune system, including T and B cells, but also by the innate immune cells, including dendritic cells (DCs), macrophages, mast cells, and natural killer (NK) cells. In addition, IL-10 can be induced in T_H_1 and T_H_17 cells by chronic inflammation as a system of feedback regulation. In this review, we focus on the molecular mechanisms underlying *IL10* gene expression in adaptive immune cells and summarize the recent progresses in epigenetic and transcriptional regulation of the *IL10* gene. Understanding the transcriptional regulatory events may help in the development of new strategies to control inflammatory diseases.

## Genetic association of IL10 with inflammatory diseases

Recent genome-wide association studies (GWAS) have demonstrated tight association of polymorphisms in the genes encoding *IL10* with systemic lupus erythematosis (SLE) (Gateva et al., 2009) and Bechet's disease (BD) (Mizuki et al., 2010; Remmers et al., 2010). BD is a genetically complex disease characterized by recurrent inflammation affecting urogenital mucosa, eye, and skin. Allelic imbalance of the rs158111 variant in pre-mRNA associates with expression of the *IL10* gene. The disease associated haplotype results in the reduction of the pre-mRNA transcript and Interleukin-10 (IL-10) production in mononuclear cells activated with lipopolysaccharide (LPS), suggesting that a genetic predisposition for low IL-10 production is a risk factor for BD (Remmers et al., 2010). Polymorphisms in the *IL10* gene region have been reported to be associated with ulcerative colitis (UC) (Franke et al., 2008), type I diabetes (Barrett et al., 2009), and severe juvenile rheumatoid arthritis (Crawley et al., 1999), and mutations in the genes encoding the subunits of the IL-10R were found in patients with inflammatory bowel disease (IBD) (Glocker et al., 2009). These observations strongly implicate IL-10 as an important regulator of the human immune system. A wide variety of cells are known to produce IL-10, but it remains unclear which cell type(s) is the major contributor to immune regulation. Therefore, it is important to better understand the source and the regulatory role of IL-10.

## The *in vivo* identification of IL-10 expression by use of reporter mice

**Figure 1:**
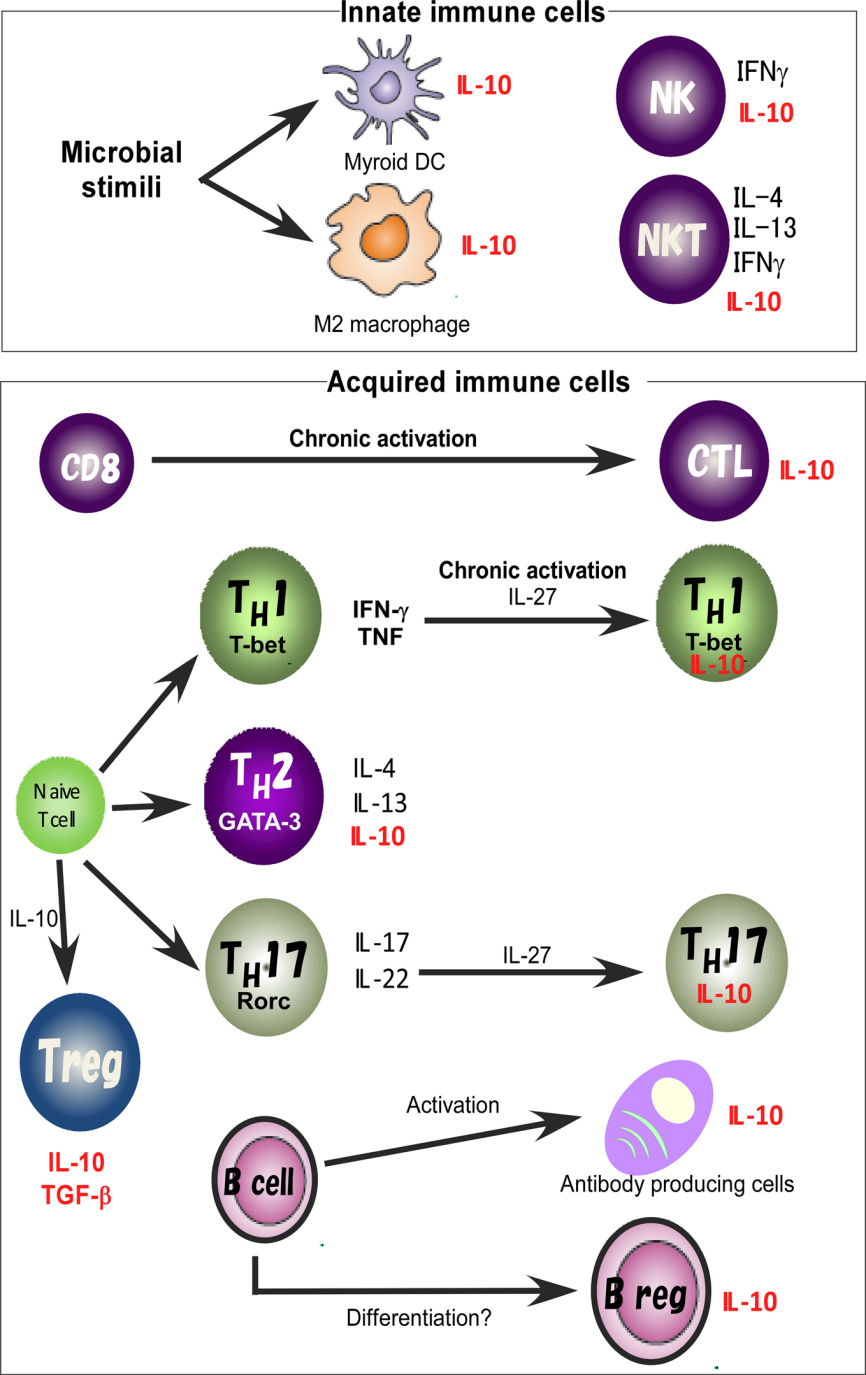
**IL-10 expression in the immune system.** IL-10 is expressed by M2 macrophages and myeloid DCs. Treg, TH1, TH2, and TH17 cell subsets share the ability to produce IL-10. IL-10 regulates the function and/or IL-10 production of Treg cells. IL-10 production by TH1 cells is induced in chronically infected mice with parasites infection and in response to high-dose antigenic stimulation. IL-27 effectively blocks IL-17 production and induces the production of IL-10. Activated B cells and Bregs are also a key B cell subset responsible for IL-10 mediated regulatory function.

For identification of the cellular sources and the role of IL-10, several reporter mouse strains have been established as useful detection tools to track *in vivo* expression of IL-10 (Bouabe, 2012). These reporter strains often provide critical insight into the expression of IL-10 in various cell types and cell type-specific function. IL-10eYFP mice and IL10-IRES-EGFP mice are classical versions of such IL-10 reporter mice (Calado et al., 2006; Kamanaka et al., 2006; Neves et al., 2010; Bouabe et al., 2011), and reporter activity in these lines has been detected only in CD4 T cells after robust stimulation. Thus these lines have a relatively insensitive limit of detection of IL-10-driven expression of autofluorescent proteins. Improved versions of the reporter mice, IL10-IRES-eGFP-BGHpA mice, IL10Venus mice and IL10-Thy1.1-SV40pA BAC transgenic mice revealed steady expression of IL-10 in a large fraction of CD4^+^ T cells, including Treg cells and NKT cells, CD19^+^B220^low^ B cells, CD19^+^ CD138^+^plasma cells, and in a very small subset of CD11b^+^ macrophages, CD11c^+^ dendritic cells (DCs), and NK1.1^+^ NK cells (Maynard et al., 2007; Madan et al., 2009; Atarashi et al., 2011). Accumulating evidence thus indicates that IL-10 is secreted by a wide variety of cells, such as helper and regulatory T cells, NKT cells, NK cells, regulatory B cells, macrophages, DCs, and monocytes, all of which may contribute to its immunoregulatory role (Figure 1).

## IL-10 production by T cells

IL-10 is one of the key cytokines to down-regulate a variety of inflammatory responses mediated by lymphoid and myeloid cells (Berg et al., 2001). IL-10 was originally identified as cytokine synthesis inhibitory factor (CSIF). It was secreted from type 2 T-helper cells (T_H_2 cells) and suppressed the differentiation and effector functions of Th1 cells (Fiorentino et al., 1989). It's inhibitory function was explained by its ability to suppress the production of pro-inflammatory cytokines such as IL-12 and TNF by DCs and macrophages, and to down regulate the expression of MHC II and the costimulatory molecules CD80 and CD86 on antigen presenting cell (APCs), thereby resulting in the subsequent inhibition of T cell activation (Fiorentino et al., 1991; Berg et al., 2001). The *in vivo* importance of IL-10 in controlling inflammatory responses was originally recognized based on observations made in IL-10-deficient mice, which mount exaggerated T_H_1 cell responses and develop spontaneous chronic enterocolitis in response to normal gut flora (Kuhn et al., 1993). IL-10-deficient mice die from spontaneous colitis (Kuhn et al., 1993), and this phenotype is partly retained even in mice lacking IL-10 only in T_reg_ cells (Rubtsov et al., 2008). In the colitis case, natural regulatory T (nT_reg_) cells would be responsible for IL-10 production in response to the microflora. On the other hand, the type 1 regulatory T (Tr1) cell is a different type of T_reg_ cell. Tr1 cells are characterized by their low proliferative capacity and their high levels of IL-10 secretion (Groux et al., 1997). Consistent with IL-10's role in suppressing inflammation, immunization of IL-10 deficient mice with myelin antigens resulted in enhanced neuroinflammation with loss of recovery from experimental autoimmune encephalomyelitis (EAE), a mouse model for human multiple sclerosis (MS) (Bettelli et al., 1998). IL-10 therefore plays an important role in regulating overactive responses that would otherwise result in autoinflammatory disease.

## Plasticity of IL-10 production by T cells

The differentiation of T_H_1, T_H_2, and T_H_17 cells is regulated by distinct signaling pathways. Despite differences in their development, these T cell subsets share the ability to make IL-10, which can be involved in the regulation of immune responses (Figure 1). The mechanism of silencing IL-10 production in T_H_1 cells was originally thought to be stable and immutable, but it is now clear that T_H_1 cells can produce IL-10 under certain conditions. IL-10-producing T_H_1 cells have been shown, on the one hand, to limit immunopathology during *Toxplasma gondii* infection (Shaw et al., 2006; Jankovic et al., 2007), but on the other hand, to attenuate protective immunity to *Leishmania major* (Anderson et al., 2007). IL-10 production by T_H_1 cells has also been reported in animals chronically infected with these parasites and in response to high-dose antigenic stimulation. Such hyperactivation of T cell receptor (TCR)-mediated signaling leads to sustained phosphorylation of ERK1 and ERK2 MAP kinases, resulting in plasticity of IL-10 production in T_H_1 cells (Saraiva et al., 2009). Continuous and excess antigen stimulation under T_H_1 skewing conditions enhances the plasticity of IL-10 production (Motomura et al., 2011), and such IL-10 production from T_H_1 cells may be an effective fail-safe mechanism to maintain immune homeostasis.

The presence of T_H_17 subsets with regulatory functions correlates with their ability to produce IL-10 (Fitzgerald et al., 2007; McGeachy et al., 2007; Stumhofer et al., 2007; Saraiva et al., 2009). IL-27 added to the cultures under T_H_17 skewing condition in the presence of TGF-β and IL-6 effectively blocks IL-17 production and induces the production of IL-10. The ability of IL-27 to induce IL-10 is important to suppress T_H_17 cell–mediated autoimmunity (Fitzgerald et al., 2007; Diveu et al., 2009). Moreover, addition of IL-27 under T_H_1 skewing conditions also induced the expression of IL-10. This activity was specific in that it had no suppressive effect on the ability of the cells to secrete IFN-γ (Batten et al., 2008). On the other hand, *in vitro* restimulation of T cells from mice with EAE in the presence of IL-23 generates cells able to efficiently transfer the disease, whereas restimulation in the presence of TGF-β and IL-6, with or without IL-23, generates nonpathogenic IL-10-producing cells (Fitzgerald et al., 2007). IL-23-induced T_H_17 cells are pathogenic and probably more responsive to IL-27-induced upregulation of IL-10. Therefore, IL-23 and IL-27, which may derive from different subsets of DC (Gafa et al., 2006; Kinnebrew et al., 2012), control the direction of T cell responses to pathogenesis or resistance. In the case of humans, IL-1β can modify the capacity of pathogen-induced human T_H_17 cells to produce either IFN-γ or IL-10 (Zielinski et al., 2012). Recently, DC and innate cell-derived IL-27 was reported to induce IL-10 production by CD8^+^ cytotoxic lymphocytes (CTLs) during viral infection (Sun et al., 2011).

In many cases, DCs possess unique functions to control the plasticity of T cell function, and the lectin-like receptors (LLR) on DCs contribute to alteration of the quality of the T cell response. Indeed, activation of DCs with Dectin-1 results in polarization into a T_H_17 response (Gringhuis et al., 2009), but Dectin-1 has also been reported to induce IL-10 production by DCs (Rogers et al., 2005). Dectin-1 in conjunction with TLR2 elicits DCs capable of inducing T_reg_ responses (Dillon et al., 2006). Another LLR, DC-SIGN, leads to not only a T_H_2 response (Geurtsen et al., 2009) and but also to T_reg_ differentiation (Zhou et al., 2010). The scavenger receptor, DC-asialoglycoprotein receptor (DC-ASGPR) leads to generation of antigen specific suppressive IL-10-producing T cells via the induction of IL-10 production by DCs (Li et al., 2012). These results and others make it abundantly clear that the regulation of T cell subset differentiation and cytokine production is complex and occurs at multiple levels.

## Transcriptional regulation of the *IL10* gene in T cells

**Figure 2:**
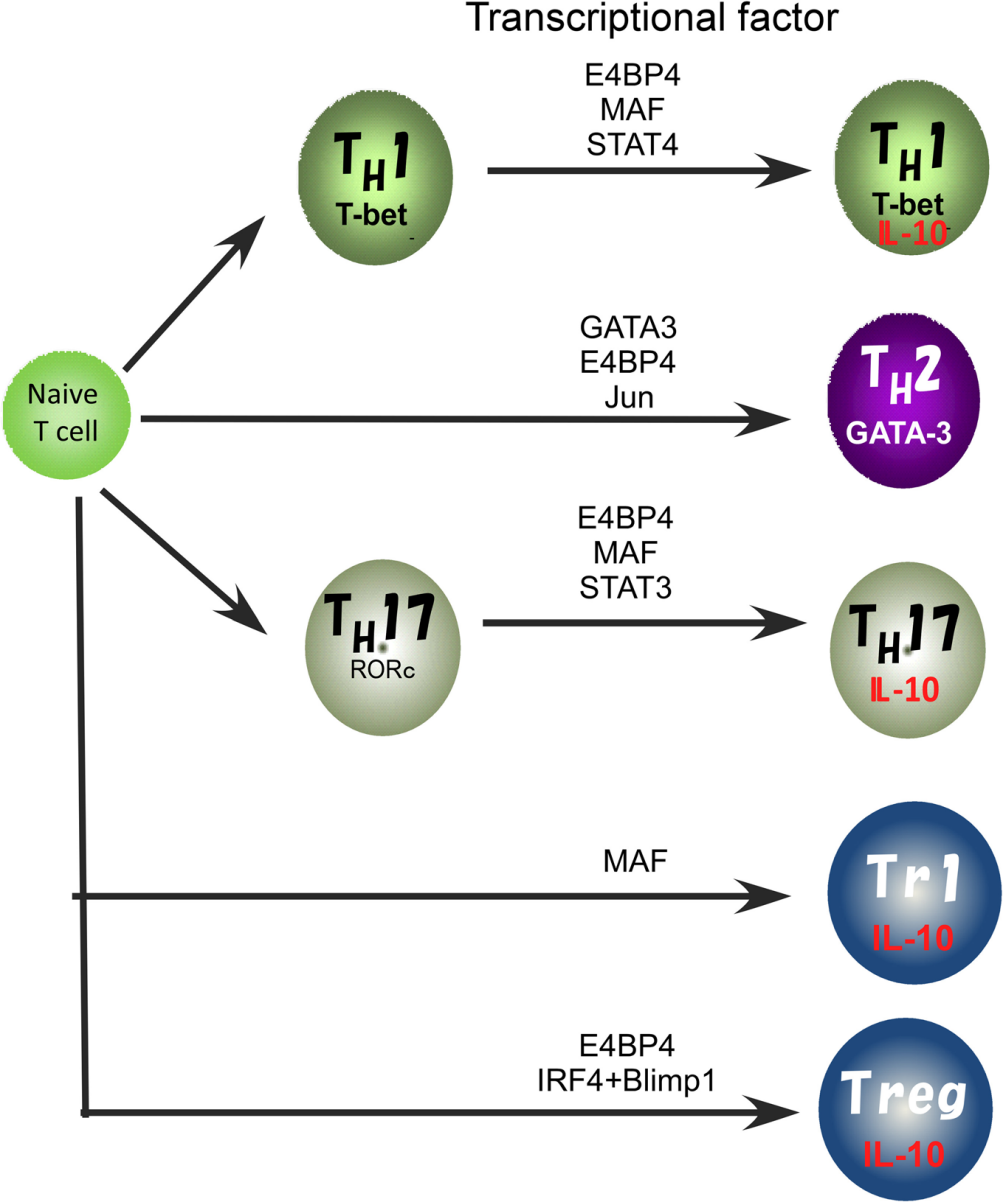
**Transcriptional factors regulating the IL10 gene expression in Treg, TH1, TH2, and TH17 cell subsets**.

In previous studies, involvement of various transcription factors in activation of the *IL10* gene has been proposed (Figure 2). The T_H_2 master regulator, GATA3 controls IL-10 expression in T_H_2 cells through initiating changes of the chromatin structure at the *IL10* locus (Shoemaker et al., 2006; Chang et al., 2007). Indeed, Gata3 deficiency cause a loss of IL-10 expression in T_H_2 cells (unpublished data). GATA3 binds to the *IL10* promoter, but GATA3 alone does not activate the promoter activity (Shoemaker et al., 2006), supporting the idea that GATA3 may determine the transcriptional permissibility of the chromatin structure in the *IL10* locus. However, in the case of other T cell types such as T_H_1 and T_H_17 that express a quite low level of GATA3, other factors would be necessary to replace its function to induce high levels of IL-10 expression (Jankovic et al., 2007; Saraiva et al., 2009).

We recently reported that E4BP4 has multiple functions in cytokine gene regulation and is an essential transcriptional factor to regulate IL-10 production not only in T_H_2, NKT, and Treg cells, but also to allow plasticity of IL-10 production in T_H_1 and T_H_17 cells (Motomura et al., 2011). E4BP4 was originally identified as a negative regulator of the mammalian circadian oscillatory system by virtue of its antagonistic binding to the same DNA regulatory sequences as a member of the PAR family of bZIP transcription factors (Cowell, 2002). The deletion of the *E4bp4* gene preferentially affected NK cell development (Gascoyne et al., 2009; Kamizono et al., 2009). By contrast, the proportion of conventional helper T cell lineages that developed in the culture conditions for T_H_1, T_H_2, iT_reg_, and T_H_17 cell induction was not affected in the *E4bp4* deficient mice. E4BP4 can induce IL-10 production under Gata3 deficient T_H_1 conditions, suggesting that GATA-3 is dispensable for plasticity of IL-10 production by T_H_1 cells. E4BP4 bound to intron 4 and the 3′ non-coding region of the *IL10* locus and activated the histone code in the *IL10* locus, as indicated by the finding that H3K9 methylation and H3K14 acetylation were completely abolished in *E4bp4*-deficient T_H_2 cells. These findings indicate that E4BP4 is an epigenetic regulator to control the permissive status of the *IL10* locus (Motomura et al., 2011).

Another T_H_2 cell-dominant transcription factor, MAF, can bind to the *IL10* promoter and plays a role in the regulation of IL-10 production in mouse macrophages stimulated with LPS and IL-4 (Cao et al., 2005). MAF is detectable in T_H_1, T_H_2 and T_H_17 cells, where its expression coincides with IL-10 production. IL-27 induces the expression of MAF along with IL-21 and the costimulatory receptor ICOS, and these ultimately act coordinately to promote differentiation of IL-10-producing Tr1 cells (Pot et al., 2009). Moreover, induction of MAF expression in T_H_1 and T_H_17 cells depends on ERK activation, as does IL-10 expression (Saraiva et al., 2009). These reports suggest that MAF may be a common regulator for IL-10 production in both the innate and the adaptive immune systems. However, MAF alone is not sufficient to induce *IL10* expression in macrophages and T cells (Cao et al., 2005; Motomura et al., 2011), thus the role of MAF in IL-10 production is still controversial.

Distinct mechanisms seem to regulate the expression of *IL10* in the innate and acquired immune systems. NF-κB activation is a major contributor to IL-10 production in macrophages, and the NF-κB p65 subunit binds to the *IL10* locus 4.5 kb upstream of the transcription start site (Saraiva et al., 2005). This finding is consistent with the report that IKK2-deficient mice show a defect in IL-10 production by LPS-stimulated macrophages. On the other hand, ERK signaling is required for optimal IL-10 induction in innate immune cells as well as T cells (Agrawal et al., 2003; Kaiser et al., 2009; Saraiva et al., 2009). ERK activation leads to the binding of AP-1 to the *IL10* locus through the cooperative function of Fos/Jun family proteins. Studies have suggested a role for Jun proteins in regulating *IL10 expression* in T_H_2 cells, but not in T_H_1 cells, and this is explained by their binding to a regulatory element located 6.45 kb downstream of the *IL10* transcription start site (Jones and Flavell, 2005; Wang et al., 2005). This report addresses the idea that ERK and MAF are required for *IL10* induction as common regulators in various cell types (Saraiva and O'Garra, 2010).

STAT proteins are reported to be another mechanism regulating IL-10 expression by both macrophages and T cells. In T cells, the induction of IL-10 by IL-27 seems to depend on both STAT1 and STAT3 (Stumhofer et al., 2007; Batten et al., 2008; Xu et al., 2009). STAT3 is also involved in IL-6-induced IL-10 expression (Stumhofer et al., 2007). Moreover, STAT3 is responsible for the IL-27-mediated IL-10 induction instead of the inhibition of T_H_17 cell differentiation. The binding of STAT3 to the *IL10* promoter in human cell lines induced *IL10* transcription though STAT3-dependent promoter activation in conjunction with IFN-α induced IRF1 activation (Ziegler-Heitbrock et al., 2003). STAT4, which is important for the expression of IFN-γ, also regulates IL-10 production in T_H_1 cells (Saraiva et al., 2009), and STAT4 was also reported to have a role in inducing its expression in NK cells (Grant et al., 2008). However, the STATs are also important in the differentiation process of T_H_ cell subsets, thus it remains unclear whether the function of STATs in IL-10 production is direct or indirect.

Recently, the coordinated activity of IRF4 and Blimp-1 has been reported to be critical for regulation of IL-10 production by Treg cells (Cretney et al., 2011). Blimp-1 is a transcriptional repressor well known for its role in promoting the differentiation of plasma cells (Nutt et al., 2008) but is also required for the maintenance of T cell homeostasis. Mice lacking Blimp-1 specifically in T cells accumulate activated T cells and develop immune pathology, including colitis and lung inflammation (Kallies et al., 2006; Martins et al., 2006), suggesting that Blimp-1 has a critical role in Treg cell function. Indeed, Blimp-1 is required for IL-10 production and high ICOS expression in mature effector Treg cells. Blimp-1 is known to be preferentially expressed in T_H_2 cells that produce high levels of IL-10, and IL-10 production by CTLs is regulated by a Blimp-1 dependent mechanism (Sun et al., 2011). Therefore, Blimp-1 is an important regulator of IL10 expression.

## IL-10 production by B cells

IL-10- producing B cells play an important role in controlling autoimmunity, such as in EAE, an animal model of MS, and in a systemic lupus erythematosus (SLE)-like disease that develops in the Lyn-deficient mouse and during murine cytomegalovirus (MCMV4) infection (Fillatreau et al., 2002; Madan et al., 2009; Scapini et al., 2011). This idea has its origins in the discovery that B cell deficient μMT mice cannot recover from EAE (Wolf et al., 1996), and the identification of a regulatory role of IL-10 producing B cells in EAE lead to the realization of the importance of this type of B cell (Fillatreau et al., 2002). Furthermore, the importance of IL-10–producing B cells in controlling chronic inflammatory diseases has also been demonstrated in collagen-induced arthritis and chronic intestinal inflammation (Mizoguchi et al., 2002; Mauri et al., 2003). On the other hand, IL-10 production by B cells has been shown to prevent protective immunity to infection with *Salmonella* Typhimurium (Neves et al., 2010) and to decrease MCMV4-specific CD8^+^ T cell responses (Madan et al., 2009).

IL-10 is secreted from several B cell subsets that can be distinguished by cell surface phenotype. Regulatory B cells (Bregs) are considered as a key B cell subset responsible for IL-10 mediated regulatory function (Fillatreau et al., 2002; Mizoguchi et al., 2002; Mauri et al., 2003). However, there is no precise marker that exclusively defines the Breg (Mauri and Bosma, 2012). Transitional 2 marginal zone precursor (T2-MZP) B cells (CD19^+^CD21^hi^CD23^hi^CD24^hi^IgD^hi^IgM^hi^CD1^hi^) appear to be the most likely candidate for being Breg cells. T2-MZP B cells isolated from arthritic mice produced copious amount of IL-10 after stimulation with collagen type II antigen in conjunction with anti-CD40 antibody. Transfer of T2-MZP B cells suppressed the development of CIA (Evans et al., 2007). T2-MZP B cells also suppress other autoimmune diseases including antigen-induced arthritis (AIA) and lupus (Inoue et al., 2006; Carter et al., 2011), and Schistosoma mansoni infection generates IL-10 producing T2-MZP B cells with regulatory function (Amu et al., 2010). However, T2-MZP B cells do not fully satisfy of the complete phenotype of Breg cells, because T2-MZP B cells also contain the largest fraction of transitional immature B cells.

CD5^+^ B1 B cells are also known to be distinct source of IL-10 (O'Garra et al., 1992), and they also have an immunoregulatory function by killing of CD4 T cells by FasL/Fas-dependent mechanisms (Lundy and Fox, 2009). B1 B cells also play a role in protection from colitis, but their protective role is not due to the production of IL-10, instead IgM and IgA are responsible for the protection (Shimomura et al., 2008).

## IL10 regulation in breg cells

In studies to understand the mechanisms underlying control of IL10 expression in B cells, the importance of TLRs has been emphasized. *In vitro* stimulation with LPS, together with PMA and ionomycin, promotes the development of IL-10 producing B cells. There is a rare population expressing CD5 and CD1d^hi^ termed IL-10 producing B cells (B10 cells) that suppresses oxazolone-induced contact hypersensitivity (Yanaba et al., 2008). Stimulation of B cells *in vitro* with LPS induces the expression of IL10 in plasma cells (CD19^+^CD138^+^), but only very low levels of IL-10 in the B10 population (Neves et al., 2010). On the other hand, TLR2 and TLR4 signaling predominantly controls IL-10 production in MZ B cells, but not in follicular B cells (Gray et al., 2007). The importance of IL-10 from B cells is confirmed in mice lacking TLR2, TLR4 or *Myd88*, a signaling molecule downstream of TLRs, which develop a chronic form of EAE (Lampropoulou et al., 2008), and expression of MyD88 is required for IL-10 production and inhibitory function of B cells. The importance of TLR9, which is a receptor for CpG, has also been proposed in regulating IL-10 production by B cells (Barr et al., 2007). However, TLR9- deficient B cells can still inhibit EAE, suggesting a redundant role of TLR9 in Breg cells (Lampropoulou et al., 2008).

Multiple studies have indicated an essential function of the CD40-CD154 interaction for the activation of Breg cells. Mice lacking CD40 on B cells develop severe EAE, with increased induction of encephalitogenic T_H_1 and T_H_17 responses, and have a profound decrease in IL-10 production (Mizoguchi et al., 2000). Furthermore, administration of an agonistic antibody against CD40 improves arthritis by the provision of IL-10 (Mauri et al., 2003; Evans et al., 2007) and IL-10 mediated T_H_1 inhibition (Mauri et al., 2000).

Several previous studies have suggested that Bregs require signaling through the B cell receptor (BCR) for their activity. Indeed, mice lacking the BCR co-receptor molecule CD19 develop a severe EAE, suggesting the importance of the BCR in the generation of Bregs (Sato et al., 1996; Yanaba et al., 2008). A major component downstream of BCR signaling is intracellular Ca^2+^ (Feske, 2007). Mice lacking the Ca^2+^ channel molecules, STIM1 and STIM2, develop an elaborated EAE and low numbers of IL-10 producing Bregs (Matsumoto et al., 2011). These mice showed normal B cell development and antibody responses, however, they have a defect in the activation of nuclear factor of activated T cells (NFAT). Inconsistently, mice with an NFATc1 deficiency display an increased number of IL-10 producing B cells and development of milder EAE (Bhattacharyya et al., 2011). Therefore, at present, it remains unclear which signaling pathway is the major one.

## Conclusion

Recent GWAS have demonstrated tight association of polymorphisms in the gene encoding *IL10* with several inflammatory diseases, indicating importance of understanding the source and the regulation of IL-10. IL-10 is expressed by many acquired immune cells including, T_H_1, T_H_2 and T_H_17 cells, Treg cells, CD8^+^ T cells and B cells. In this review, we have summarized the current view of *IL10* regulation in these T cells and B cells. *IL10* expression in different T cell subsets is regulated by a complex of multiple transcriptional factors, such as GATA-3, E4BP4, MAF, Blimp1, and so on, and these multiple levels of regulation occur at independent differentiation stages in accordance with certain rules that are just beginning to be understood. Therefore, the expression of the *IL10 gene* has the flexibility and plasticity. This plasticity is sometimes controlled by continuous antigen stimulation or by a particular cytokine environment, such as DC derived IL-27, and selectively occurs in inflammatory type of helper T cells, T_H_1 and T_H_17 cells. On the other hand, the importance of IL-10 from B cells is proposed in several autoimmune and inflammatory disease models. Similar to T cell, a complex of multiple signaling pathways, including BCR and CD40-CD40L pathways, is required for the generation of Bregs and the induction of IL-10 in B cells. However, it remains virtually unknown at present which signaling pathway is the major one and how *IL10* is transcriptionally regulated. There may be critical transcriptional regulatory machinery that could be specific to certain cell types and this machinery may be turned on by specific signals in certain diseases. Therefore, it will be quite important to understand the meaning of the SNPs that associate with several inflammatory diseases and the molecular mechanisms underlying transcriptional regulation of the *IL10* gene. Moreover, this approach may lead to the development of innovative therapeutic strategies for controlling these diseases.

### Conflict of interest statement

The authors declare that the research was conducted in the absence of any commercial or financial relationships that could be construed as a potential conflict of interest.
